# Fluorescence Resonance Energy Transfer for Drug Loading Assessment in Reconstituted High-Density Lipoprotein Nanoparticles

**DOI:** 10.3390/ijms26073276

**Published:** 2025-04-01

**Authors:** R. Max Petty, Luca Ceresa, Emma Alexander, Danh Pham, Nirupama Sabnis, Rafal Fudala, Andras G. Lacko, Raghu R. Krishnamoorthy, Zygmunt Gryczynski, Ignacy Gryczynski

**Affiliations:** 1 Department of Pharmacology and Neuroscience, North Texas Eye Research Institute, Graduate School of Biomedical Science, University of North Texas Health Science Center, Fort Worth, TX 76107, USA; roland.petty@my.unthsc.edu (R.M.P.); raghu.krishnamoorthy@unthsc.edu (R.R.K.); 2 Department of Physics and Astronomy, Texas Christian University, Fort Worth, TX 76109, USA; luca.ceresa@tcu.edu (L.C.); e.l.alexander@tcu.edu (E.A.); danh.pham@tcu.edu (D.P.); z.gryczynski@tcu.edu (Z.G.); 3 Department of Physiology and Anatomy, Graduate School of Biomedical Sciences, University of North Texas Health Science Center, Fort Worth, TX 76107, USA; nirupama.sabnis@unthsc.edu (N.S.); rafal.fudala@unthsc.edu (R.F.); 4 Department of Microbiology, Immunology, and Genetics, School of Biomedical Sciences, University of North Texas Health Science Center, Fort Worth, TX 76107, USA; andras.lacko@unthsc.edu

**Keywords:** nanoparticles, FRET, fluorescence lifetime, drug delivery, lipoproteins

## Abstract

Reconstituted high-density lipoprotein nanoparticles (NPs), which mimic the structure and function of endogenous human plasma HDL, hold promise as a robust drug delivery system. These nanoparticles, when loaded with appropriate agents, serve as powerful tools for targeted drug delivery. The fundamental challenge lies in controlling and estimating the actual drug load and the efficiency of drug release at the target. In this report, we present a novel approach based on enhanced Förster Resonance Energy Transfer (FRET) to assess particle load and monitor payload release. The NPs are labeled with donor molecules embedded in the lipid phase, while the spherical core volume is filled with acceptor molecules. Highly enhanced FRET efficiency to multiple acceptors in the NP core has been observed at distances significantly larger than the characteristic Förster distance (R_0_). To confirm that the observed changes in donor and acceptor emissions are a result of FRET, we developed a theoretical model for nonradiative energy transfer from a single donor to multiple acceptors enclosed in a spherical core volume. The load-dependent shortening of the fluorescence lifetime of the donor correlated with the presence of a negative component in the intensity decay of the acceptor clearly demonstrates that FRET can occur at a large distance comparable to the nanoparticle size (over 100 Å). Comparison of theoretical simulations with the measured intensity decays of the donor and acceptor fluorophores constitute a new method for evaluating particle load. The observed FRET efficiency depends on the number of acceptors in the core, providing a simple way to estimate the nanoparticle load efficiency. Particle disintegration and load release result in a distinct change in donor and acceptor emissions. This approach constitutes a novel strategy for assessing NP core load, monitoring NP integrity, and evaluating payload release efficiency to target cells. Significants: In the last decade, nanoparticles have emerged as a promising strategy for targeted drug delivery, with applications ranging from cancer therapy to ocular neurodegenerative disease treatments. Despite their potential, a significant issue has been the real-time monitoring of these drug delivery vehicles within biological systems. Effective strategies for monitoring NP payload loading, NP integrity, and payload release are needed to assess the quality of new drug delivery systems. In our study, we have found that FRET-enabled NPs function as an improved method for monitoring these aspects currently missing from current drug delivery efforts.

## 1. Introduction

Plasma lipoproteins were proposed to function as effective drug nanocarriers nearly 40 years ago [1]. Reconstituted high-density lipoprotein (rHDL) vehicles are ideal for drug delivery due to their small size (10–40 nm), which allows them to navigate vasculature and bypass biological barriers, such as the blood–brain barrier, without eliciting an immunogenic response. The presence of the apolipoprotein A-I (Apo A-I), which allows the rHDL to deliver its payload through the scavenger receptor class B type 1 (SR-B1) specifically, offers a novel drug delivery mechanism not found on conventional nanoparticles [2]. Subsequently, research over the last several decades has primarily focused on developing and evaluating synthetic or reconstituted HDL nanoparticles (NPs) as a “magic bullet” approach for drug delivery, especially for cancer therapeutics [2,3,4,5]. Nevertheless, no lipoprotein-based drug formulation has reached clinical applications so far. The main concerns delaying the progress of lipoprotein-based nanostructures toward practical applications include the lack of simple methods to evaluate particle load, the absence of practical techniques to monitor long-term particle stability and drug delivery and release to target tissues in the body, plus the challenges in scaling up the preparation process to commercial or manufacturing levels [2,3,4,5,6]. Although various novel preparation methods utilizing peptide mimetics of Apo A-I show potential to significantly accelerate the development and scaling up of therapeutic rHDL formulations, we still lack robust approaches for quick load estimation and methods to monitor particle stability and load release [7,8].

In recent decades, fluorescence has emerged as a promising technology for tracking and monitoring nanoparticle distribution in tissues. Using optical or near-IR fluorescent markers or fluorescent drugs makes the visualization and tracking of markers and NPs in tissues feasible [9,10,11,12,13,14,15,16,17,18,19]. However, challenges remain in estimating NP load, load distribution within the NP, and importantly, monitoring NP integrity during storage and during the drug delivery process. The optimal NP size range is 10–40 nm, presenting significant challenges in accurately estimating NP payload and stability. The fundamental problem is the lack of a physical process or phenomenon that allows monitoring sizes and interactions within the 10–50 nm range. This range is too large for the typical nonradiative range of Förster Resonance Energy Transfer (FRET) [20,21,22,23,24] and too small for super-resolution approaches [25,26]. The critical limitation of the FRET process is an efficient interaction range that depends on the donor-acceptor pair, which for the most suitable pairs is still below 10 nm. While super-resolution methods may theoretically reach slightly below 50 nm, the imaged sample region is very small (comparable to a single cell), making them unsuitable for practical tissue or organ imaging; in addition, these approaches require highly sophisticated instrumentation.

A few years ago, we proposed an approach that demonstrated the possibility of significantly extending the effective range for FRET beyond a range of 10 nm [27]. By positioning several acceptors on the surface of a globular protein, we showed that efficient energy transfer could occur at distances over 15 nm. The proposed rHDL nanoparticles present an interesting system that can easily accommodate many drug molecules or chromophores (over 10 molecules) in a small, confined volume, which could serve as a ’super acceptor’ to an externally positioned donor. The FRET efficiency will depend on the payload (the number of acceptors in the core) and will be highly sensitive to NP integrity. Such a setup would constitute a simple way for monitoring NP load during preparation and storage and NP integration and payload release during delivery. However, investigations in complex systems like tissues or NPs are frequently affected by intrinsic scattering and/or autofluorescence which poses a problem in interpreting experimental spectra and measured intensity decays.

In order to utilize this approach for practical applications, we need to prove that observed changes in spectra and intensity decays result from long-distance FRET (much farther than a Forster distance, R_0_) and are not the result of scattering, molecular interactions, and other artifacts. In this report, we present detailed studies of FRET between external donors and multiple acceptors loaded into the NP core. We provide theoretical considerations to support and interpret experimental results. We analyze spectral changes (decrease in donor emission accompanied by an increase in acceptor emission) and, most importantly, the changes in intensity decays of the donor and the acceptors. A decrease in donor fluorescence lifetime in the presence of an acceptor is typically considered a “gold standard” confirming the energy transfer. However, in complex systems and the presence of high scattering, experimentally measured decreases in donor lifetime could artificially be shortened by leaking scattering, interactions with the local environment, or intrinsic tissue autofluorescence. Since nonradiative energy transfer is an instantaneous yet statistical process, the intensity decay of the acceptor observed with donor excitation should reflect the intensity decay of the donor as a ’pumping’ function. This will manifest as a negative amplitude component in the intensity decay of the acceptor. Correlating such an anomalous intensity decay of the acceptor with changes in the intensity decay of the donor would provide the most direct proof that the observed changes are solely due to the FRET.

**Figure 1:**
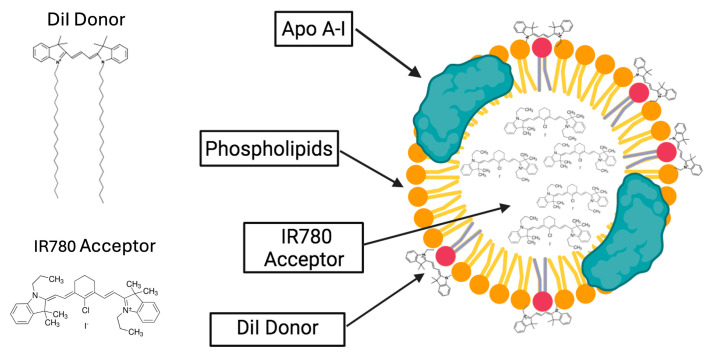
Schematic presentation of the dual fluorescent DiI-rHDL-IR780 NPs loaded with the acceptor fluorophore, IR780 iodide, and labeled with the donor fluorophore, DiI (in red), equipped with two 18:0 carbon chains inserted into the EYPC phospholipid layer (in orange) with the fluorophore moiety extended outwards from the surface of the NPs.

**Figure 2:**
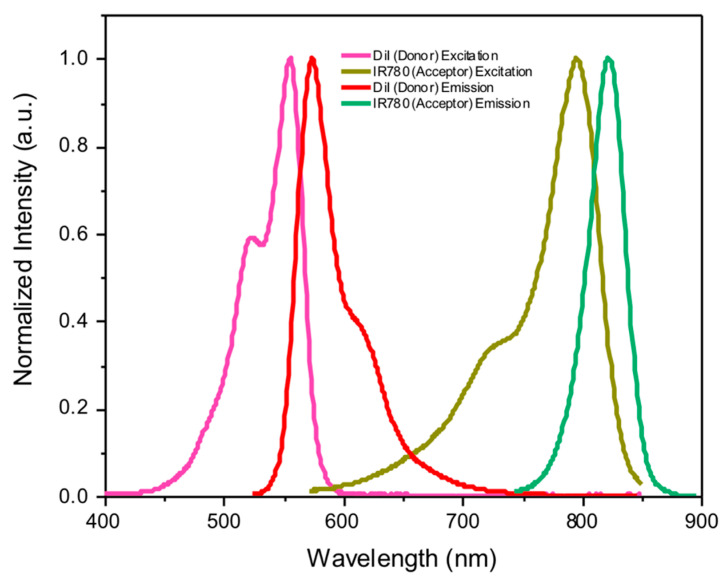
The absorption and emission spectra for free donor and acceptor fluorophores in DMSO, with limited spectral overlap at 662 nm. The calculated R_0_ value is 28 Å.

In the first part, we present a theoretical consideration of the expected results. We analyze anticipated changes in emission (in emission spectra and fluorescence lifetime for both the donor and acceptor) for a large NP system where the overall donor-acceptor separation significantly exceeds the characteristic Förster distance, R_0_ (separations over 2 R_0_). To experimentally confirm our theoretical predictions and assess payload loading, biodistribution, and payload release, we assembled rHDL nanoparticles in which IR780 iodide was loaded into the interior core of the rHDL complex, and the lipid layer on the exterior of the NP was labeled with DiI (1,1′-Dioctadecyl-3,3,3′,3′-tetramethylindocarbocyanine perchlorate), as shown in Figure 1. For NP preparations, we used a method we previously standardized [3], which consistently produces particles with an approximate diameter of 10 nm and an internal core size of about 4 nm. We maintained a constant density of DiI dye embedded in the lipid layer while increasing the IR780 payload by adjusting its concentration. The efficiencies for DiI labeling and IR780 loading in each preparation were confirmed by measuring the absorption spectra of purified NPs. As schematically presented in Figure 1, DiI molecules (donors) are embedded into the membrane with the main fluorophore moiety standing out of the membrane. The separation of DiI to the center of the NP core is at least the membrane thickness, which is in the range of 4 nm with a core radius of approximately 2 nm. The absorption and emission spectra for donor and acceptor fluorophores are shown in Figure 2 and show a large spectral separation. This results in only limited spectral overlap with an estimated characteristic Forster distance (R_0_) of approximately 2.8 nm (28 Å) for the DiI-IR780 pair, significantly less than the NP size. However, substantial spectral separation of the donor and acceptor will allow detailed analysis of observed spectral changes and, importantly, enable independent monitoring of intensity decays of the donor and the intensity decay of the acceptor with no crosstalk from donor emission. This will allow clear distinction in the intensity decays of donor and acceptor with excitation wavelength suitable for the donor. Consequently, this approach will constitute a straightforward method to monitor particle loading, particle stability, and disintegration, opening new avenues for future testing of developed nanoparticles and real-time monitoring of delivery and content release. When using more suitable FRET pairs with characteristic Forster distance, R_0_, in the range of 50 Å, the methods can be used for much larger particles in the range of 20–30 nm.

### Theoretical Considerations

*Enhanced FRET.* Over the last 50 years, there has been a dramatic increase in the application of fluorescence and FRET technologies in the biomedical sciences. FRET is a long-range dipole-dipole, through space interaction between two chromophores (one energy donor and one energy acceptor), heavily dependent on the distance (to the 6th power) between the participating chromophores. The FRET phenomenon and its applications have been described in multiple scientific papers and books [20,21,22,23,24,28]. The efficiency of the energy transfer depends on the characteristic Förster radius that is dictated by several factors, including the quantum yield of the donor, refractive index of the medium, spectral overlap between emission of the donor and absorption of the acceptor, and relative orientation of interacting dipoles (molecules) [20,21,22,23,24]. The characteristic Förster distance is typically below 50 Å, limiting the interactions to distances much below 100 Å (10 nm). FRET is now commonly used as a standard tool for measuring intramolecular distances, functioning as a ‘spectroscopic ruler’, and is a highly efficient method for assessing interactions between or within small molecular systems (e.g., proteins and/or DNA/RNA) [22]. However, larger molecular ensembles exceeding 100 Å in diameter are beyond the reach of typical FRET technology.

**Figure 3:**
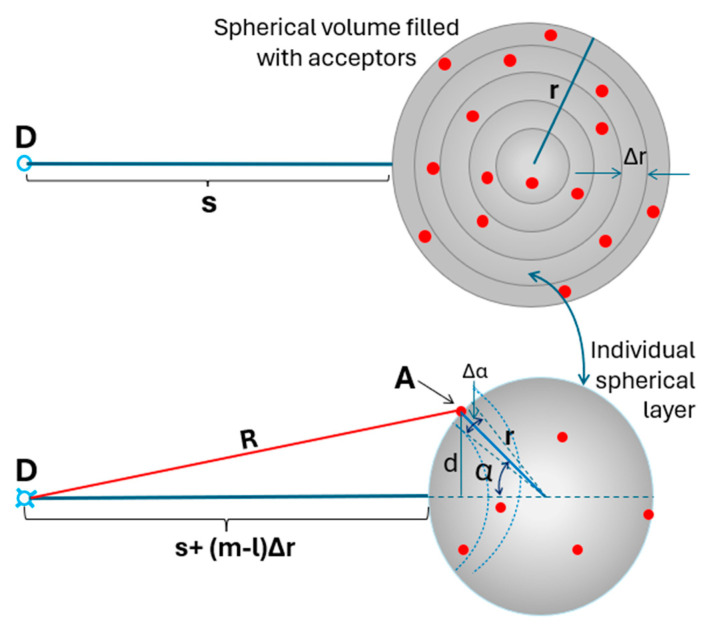
(**Top**). Onion-type representation of a spherical volume filled with n acceptors (●). (**Bottom**). A model system of a single donor, D (¤) and acceptors, A (●) randomly distributed on a spherical surface of radius r.

Previously we calculated the average transfer efficiency to the *n* acceptors randomly positioned on a small spherical surface like a globular protein [27] demonstrating that interaction can easily be extended to about 150 Å, over twice the R_0_ distance (9) just for 4–6 acceptors positioned/labeled on avidin surface. In the present approach, we are considering a spherical volume of a radius *r* randomly filled with *n* acceptors (Figure 3). Using results from our previous approach, we will calculate average FRET efficiency by dividing the volume of a sphere by a given number of spherical layers (so-called onion model—Figure 3 Top). Let us assume the volume is divided into *m* layers, each of a thickness Δ*r* = *r*/*m*. The radius of each layer is *r_l_* = *l* Δ*r*, and the volume of each layer can be described as *V_l_* = 4*πr_l_*^2^Δ*r* = *4π l l*^2^Δ*r*^3^ where *l* = 1, 2, …, *m −* 1. The sum of volumes for all layers is equal to the volume of a sphere of radius r (4*πr*^3^/3). Each layer will contain average nl=nVl43πrl3 acceptors.

For a large number of layers (large *m*) and a random distribution of acceptors, the expected FRET efficiency from a mobile donor to randomly distributed *n_l_* acceptors contained in a given layer volume, *V_l_* that can be considered as a sphere surface (Figure 3 Bottom) can be calculated as follows [27]:

(1) $$E_{l}^{T}=\frac{n_{l}k_{l}^{t}}{n_{l}k_{l}^{t}+1/\tau_{0}}=\frac{k_{l}^{T}}{k_{l}^{T}+1/\tau_{0}}$$

where $k_{l}^{T}=n_{l}k_{l}^{t}$ is the expected average transfer from a single donor to *n_l_* acceptors in each layer (*l*) that are randomly distributed on a sphere surface of radius r*_l_*:

(2) $$k_{l}^{t}=\frac{\sum_{j}{∆S}_{j}^{l}k_{j}}{4\pi r_{l}^{3}}$$

where the surface element ${∆S}_{j}^{l}=2\pi d_{j}\Delta \alpha =2\pi r_{l}\sin\alpha_{j}\Delta \alpha$, and Δ*α* is the angle element as shown in Figure 3 (Bottom) (for details see [27]).

**Figure 4:**
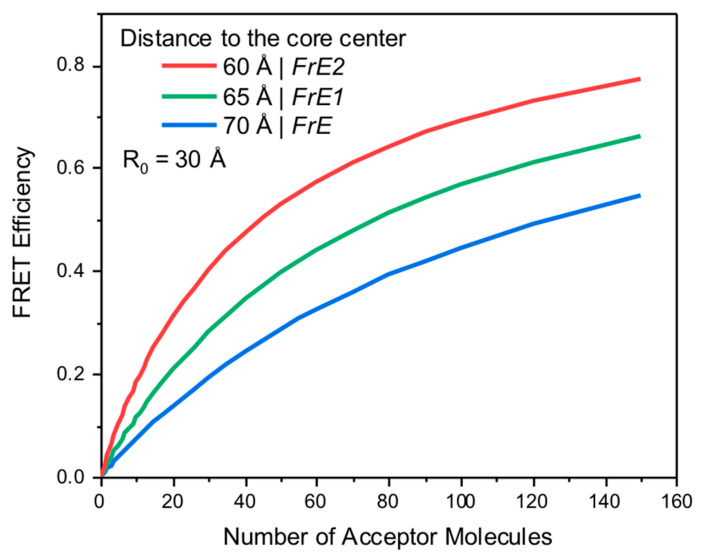
Dependence of effective FRET transfer efficiency as a function of the number of acceptors in the core for three different separations of the external donor to the center of the core: 60 Å, 65 Å, and 70 Å. The calculated R_0_ value for this simulation was 30 Å.

A total transfer to the entire volume/core will be a simple sum of transfer rates to each layer/shell. NPs prepared are approximately 10 nm in diameter with a core size of about 4 nm, as confirmed by Malvern Zetasizer using dynamic light scattering measurements. To approximate these conditions, we simulated the transfer efficiency for a system where the initial separation from the external donor to the center of the core is in the range of 60–70 Å (more than 2 times R_0_). In Figure 4, we present simulated transfer efficiencies as a function of the number of acceptors in the core for three different separations of the external donor to the center of the core: 60 Å, 65 Å, and 70 Å, assuming the R_0_ of 30 Å. It is clear that with only one acceptor randomly positioned in the core, the average FRET is too low to be experimentally measured (below 2%). However, with 5 acceptors in the core, we can detect changes, and with 10 or more acceptors, the effect should be significant in the range of 10%.

It is educational to consider the intensity decay of donors and acceptors. Observed intensity is directly proportional to the number of molecules in the excited state. The time-dependent population of donors and acceptors after delta-pulse excitation, when no energy transfer occurs, is, respectively, as follows:

(3) $$N_{D}\left(t\right)=N_{D}^{0}e^{-k_{D}t}\;\;\;\;\;and\;\;N_{A}\left(t\right)=N_{A}^{0}e^{-k_{A}t}$$

where $N_{D}^{0}\;and\;N_{A}^{0}$ are the number of excited donors and acceptors, respectively, at time 0, $k_{D},\;and\;k_{A}$ are decay rate constants for donor and acceptor, respectively. For simplicity of presentation $k_{D}\;and\;k_{A}$ represent the total (sum of the radiative and non-radiative) decay rate. In the presence of FRET, we have the following:

(4) $$N_{D}\left(t\right)=N_{D}^{0}e^{{-(k}_{D+}k_{T})t}\;\;\;\;$$

where $k_{T}$ is the energy transfer rate. Since initial lifetime of donor, τD0=1kD  the lifetime of the donor in the presence of acceptor will be τDT=1(kD+kT) . From measurements of donor lifetime, we can calculate the number of donors that transfer energy to the acceptors:

(5) $$N_{D}^{T}=N_{D}^{0}(1-\frac{\tau_{D}^{T}}{\tau_{D}^{0}})$$

$N_{D}^{T}$ represents the number of donor molecules that transfer energy to the acceptors. As energy is transferred from a single donor to a single acceptor (one donor may only transfer energy to one acceptor independently of how many acceptors are in proximity), the number of excited acceptors via energy transfer is $N_{A}^{T}=N_{D}^{T}$. For a perfect experiment, it would be beneficial to excite the donor at a wavelength where the acceptor does not absorb. This is impossible since the absorption spectrum of the donor will always overlap with the absorption spectrum of the acceptor (Figure 2). Therefore, the optimal solution is to excite the donor at a wavelength where the excitation of the acceptor is minimal. According to Equation (4), measurements of the donor lifetime in the presence of acceptors give immediate information on the efficiency of energy transfer, *E* is as follows [20,21,22,23,24]:

(6) $$E=1-\frac{\tau_{D}^{T}}{\tau_{D}^{0}}$$

Measurements of the fluorescence lifetime of the donor are considered as the most appropriate way to evaluate FRET efficiency. However, in a complex system, such as NPs randomly labeled with donors, there are multiple processes that may affect measured lifetime. This includes donor quenching by the environment, self-quenching where two or more donors are closely positioned, and importantly, high scattering of the NP solution that may lead to scattered light leaking through the emission channel, resulting in an apparent shortening of the measured fluorescence lifetime. Under normal conditions, the intensity decay of the acceptor is described by Equation (3). In the presence of FRET, a significant population of excited acceptors can be generated by an energy transfer from a donor. This time-dependent process will depend on energy transfer efficiency, relative donor and acceptor populations (relative number of excited donors as compared to the number of directly excited acceptors), and the relative fluorescence lifetimes of the donor and acceptor. In general, the time evolution of donor and acceptor populations can be described by a set of differential equations:

(7) $$\frac{dN_{D}}{dt}=-\left(k_{D}+k_{T}\right)N_{D}+A_{D}L(t)\;\;\frac{dN_{A}}{dt}=-\left(k_{A}\right)N_{A}+k_{T}N_{D}+A_{A}L(t)$$

where *N_D_* and *N_A_* are populations of the donor and acceptor, respectively, *A_D_* and *A_A_* are absorptions of the donors and acceptors at the excitation wavelength, and *L*(*t*) is the excitation pulse function. The solution of these two equation systems was originally given by Laws and Brant [29] and later by us [30]. The population decays of donor and acceptor are given by the following:

(8) $$\begin{array}{l} \;N_{D}\left(t\right)=N_{D}^{0}e^{-(k_{D}+k_{A})}\\ N_{A}\left(t\right)=B\exp\left[-\left(k_{D}+k_{ET}\right)t\right]+\left(N_{A}^{0}-B\right)exp[-k_{A}t] \end{array}$$

where $N_{D}^{0}$ and $N_{A}^{0}$ are the number of excited donors and acceptor molecules at *t* = 0 that are, in this case, proportional to $A_{D}L\left(t\right)\;and\;A_{A}L(t)$. The relative number of excited donors and acceptors is proportional to their absorptions at the excitation wavelength. The factor *B* is as follows:

(9) $$B=\frac{N_{D}^{0}k_{T}}{k_{A}-k_{D}-k_{T}}$$

**Figure 5:**
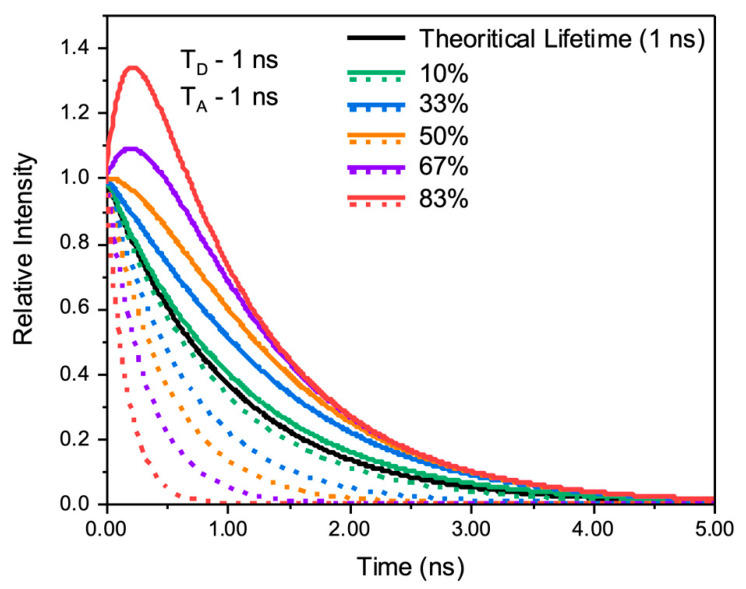
The theoretical intensity decay for dual fluorescent DiI-rHDL-IR780 NPs, where donor (dashed) and acceptor (solid) simulations are for different efficiency of energy transfer.

The decay of the donor in the presence of FRET was predicted by Equation (4) and agrees with Equation (8). The energy transfer rate (*k_T_*) contributes to donor excited state deactivation, resulting in a shortening of donor fluorescence lifetime. However, the decay of the acceptors is a much more complex function. The same energy transfer rate that deactivates donors contributes positively to the population of excited acceptors. This positive factor is directly related to the population of donors in the excited state compared to directly excited acceptors, as part of the donors decaying via energy transfer that populates the excited state of acceptors. Effectively, energy transfer contributes to the population of excited acceptors over the lifetime of the donor. This contribution via energy transfer rate should manifest as a positive component in the intensity decay of the acceptor (so-called ‘pumping’ function). The effect will depend on the FRET efficiency in a complex way. Higher transfer rates give larger contributions but in a shorter time (the lifetime of the donor gets shorter as FRET increases). In Figure 5, we present expected intensity decays for the donor (dashed lines) and acceptor (solid lines), assuming the initial populations of excited donors and excited acceptors are identical (50:50). For the simulation, we assumed lifetimes of 1 ns for the donor and the acceptor. At the initial stage of the decay, a clear increase in the acceptor population can be observed even for a small efficiency of 10%. Observing such a positive intensity decay component (pumping) in the acceptor decay would be undeniable proof of energy transfer since no other process can result in this type of change.

*Optimizing the experimental conditions.* To experimentally confirm that FRET efficiency can be used to estimate NP load, we are preparing nanoparticles according to the protocol developed earlier. With this established preparation protocol, it is relatively easy to maintain consistent NP size and external core labeling while varying the concentration of the added payload (dye). We prepared NPs with a consistent average core size (~4 nm) and lipid layer of 3.5 nm that does not vary much from prep to prep, while the payload increases accordingly.

To test our theoretical predictions, we purposely selected the donor-acceptor pair for which spectra are well separated (Figure 2). Although this significantly lowers the R_0_ for the system, it allows for precise monitoring of changes in absorption and emission spectra and, most importantly, allows independent monitoring of intensity decay of donor and acceptor with no crosstalk (emission of donor leaking into the emission of acceptor).

## 2. Results and Discussion

To test our theoretical prediction, we assembled rHDL nanoparticles in which IR780 (acceptor) was loaded into the interior core of the rHDL complex, and the lipid layer on the exterior of the NP was labeled with DiI (donor). For consecutive preparations of NPs, we varied the payload by changing the concentration of IR780 used for a given preparation while keeping the NP size and external labeling with the donor constant. We prepared NPs using concentrations of IR780 0% (donor only), 33 μM, 66 μM, 99 μM, and 330 μM, respectively. We also prepared NPs loaded with acceptor only (no donor labeling) for each concentration of acceptor and NPs labeled with donor only. NP sizes and zeta potentials were confirmed by Malvern Zetasizer measurements are presented in Table 1. For acceptor concentrations up to 100 μM, the size of prepared NPs and size distributions are consistent. For the highest acceptor concentration, we noticed a slight increase in the NP size, as we would expect due to the donor protrusion from the NP surface (Figure 1).

**Figure 6:**
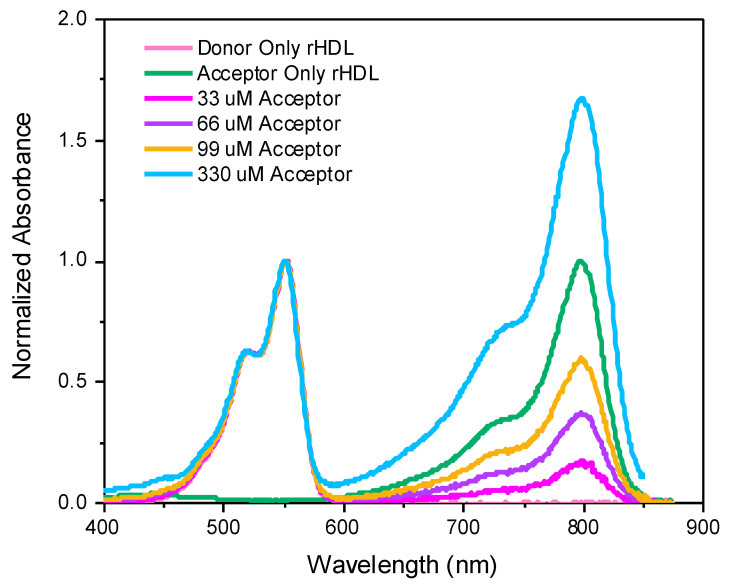
Normalized absorption spectra for dual fluorescent DiI-rHDL-IR780 NPs prepared with 4 different concentrations of loaded acceptor: 33 μM, 66 μM, 99 μM, and 330 μM of IR780. Donor (DiI) concentrations remained constant for all formulations at 214 μM. Spectra measurements for donor-only NPs, with a donor concentration of 214 μM, and acceptor-only NPs, with an acceptor concentration of 66 μM, are also included.

Figure 6 presents the absorption spectra measured for NP solutions prepared with 4 different concentrations of loaded acceptor and spectra measured for NPs with donor only and acceptor only. The concentration of solutions with different acceptor loads was adjusted to present comparable absorption at the donor absorption band. Matching different solutions with a precision better than 5% is difficult, so to allow for direct comparison, the spectra were further normalized to donor absorption. The shape of the absorption spectrum of the donors for various preparations is identical, whereas the absorption of acceptors consistently increases with a higher load. For the first couple of preparations, we observed proportional increases in the IR780 absorptions as concentration increased, however, for higher concentrations, the increase in absorption is less than expected. This may indicate that the IR780 absorption is approaching the packing capacity within the NP for concentrations over 300 μM.

**Figure 7:**
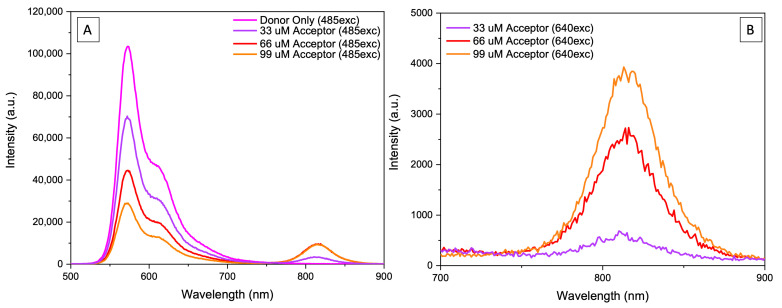
(**A**) Individual emission spectra (excitation 485 nm) of dual fluorescent DiI-rHDL-IR780 NPs as donor and acceptor., with 3 different acceptor concentrations. (**B**) Emission spectra from the 700 to 900 nm range at an excitation wavelength of 640 nm demonstrate donor emission is free from acceptor emission.

In Figure 7, we present steady-state emission spectra for NPs containing different loads when excited at 485 nm. As the load increases, the donors’ emission proportionally decreases. Initially, the acceptor emission increases until it stabilizes at a concentration of 66 μM, followed by a slight decrease at the highest concentration. This contrasts with absorption measurements, which clearly show a consistent increase in acceptor absorption as the load increases. The observed emission behavior is due to acceptor concentration-dependent self-quenching, which lowers the emission of acceptors. This was later confirmed through acceptor fluorescence lifetime measurements (presented later). The concentration-dependent self-quenching of acceptors partially compensates for the increase due to the FRET.

**Figure 8:**
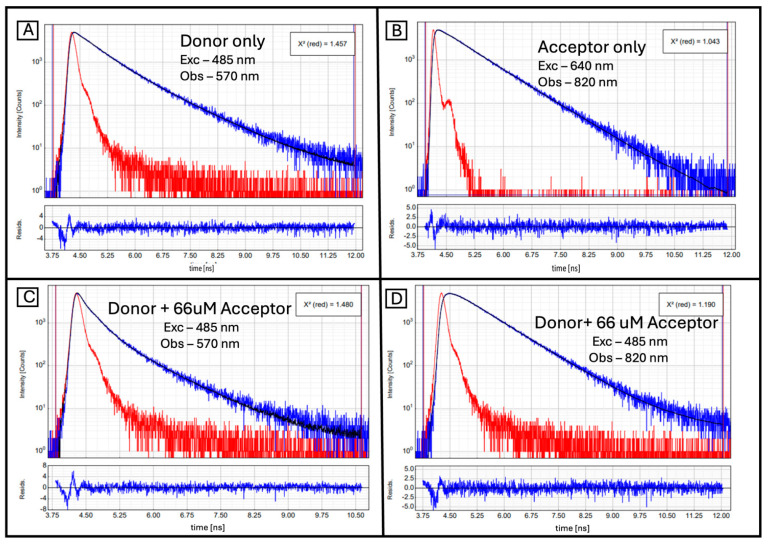
(**A**) The intensity decay and average fluorescence lifetime of the donor-only sample, with an excitation of 485 nm and observation at 570 nm. (**B**) shows the intensity decay and average fluorescence lifetime of the acceptor-only sample, with excitation at 640 nm and observation at 820 nm. (**C**) shows the intensity decay and average fluorescence lifetime of the rHDL NP with a donor in the presence of the acceptor (66 μM loading), at excitation of 485 nm and observation at 570 nm. (**D**) shows the intensity decay and average fluorescence lifetime of the rHDL NP with donor in the presence of the acceptor (66 μM loading), at excitation of 485 nm and observation at 820 nm. Red lines represent the IRF while the blue lines represent the fluorescence intensity decay of the sample.

Figure 8 presents the fluorescence intensity decays for donor-only and acceptor-only samples (top) for a selected loading concentration of 66 μM acceptor loading (different loads are presented in Supplementary Materials). The donor was excited at 485 nm, and emission was monitored at 570 nm. Acceptor-only samples were excited at 640 nm, and emission was monitored at 820 nm. Acceptor excitation at 485 nm produced a much lower signal due to the low absorbance, but both excitations (485 nm and 640 nm) exhibited practically the same intensity decay. The intensity decays for the donor-acceptor system (66 μM) were monitored at 570 nm, where only the donor emits, and at 820 nm, where the acceptor emission completely dominates. The emission of the donor is quenched by FRET, resulting in a faster intensity decay. Measured intensity decays for 33 μM, 99 μM, and 330 μM are presented in Supplementary Materials. Conversely, the intensity decay of the acceptor becomes more complex. Detailed analysis of the intensity decay parameters (fluorescence lifetimes and amplitudes) is presented in Table 2. As expected, the lifetime of the donor consistently becomes shorter as the concentration of the acceptor increases. Analysis of acceptor intensity decays reveals a lifetime component with a negative amplitude. The highest concentration (330 μM) yields a satisfactory fit with positive amplitudes. However, the lifetime of the quenched donor is significantly shorter, and energy transfer (pumping) occurs on a timescale comparable to the IRF, causing the positive component to be folded into the IRF.

**Figure 9:**
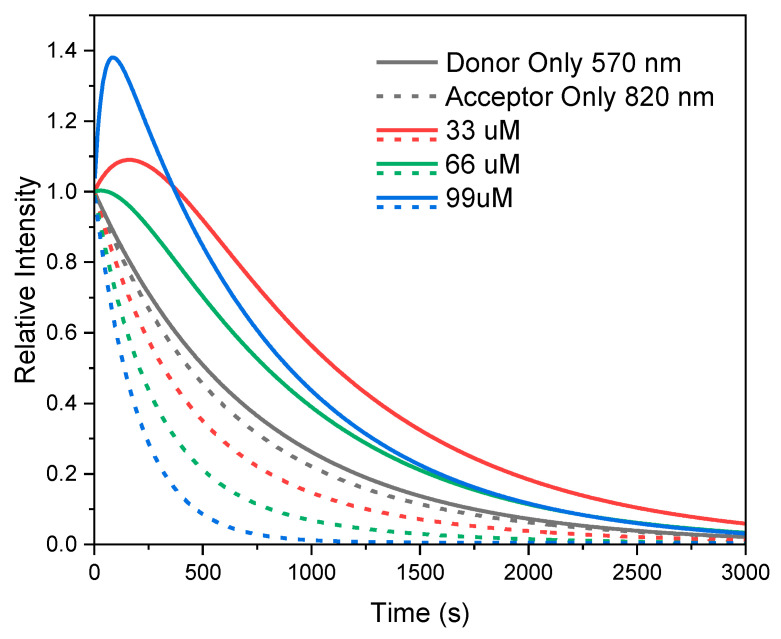
Experimental data for the intensity decay for dual fluorescent DiI-rHDL-IR780 NPs with excitation 485 nm and emission observed at either 570 nm for donor (dashed line) or 820 nm for acceptor (solid line) for different efficiency of energy transfer.

To compare experimental results to our predictions presented in Figure 4, one needs to remember that the measured intensity decays presented in Figure 8 are convolutions of the decay and IRF, shown in the logarithmic scale. To directly compare the data in Figure 8 to our predictions in Figure 4, we reconstructed intensity decays from recovered parameters in Table 2. Figure 9 presents the recovered intensity decays for 485 nm excitation, for donor-only and acceptor-only samples (dashed and solid black, respectively), for the 33 μM acceptors loading (red dashed for donor—570 nm observation and solid for acceptor—820 nm observation), 66 μM acceptor loading (green dashed for donor—570 nm observation and solid for acceptor—820 nm observation), for 99 μM acceptor loading (blue dashed for donor—570 nm observation and solid for acceptor—820 nm observation). The recovered intensity decays clearly show predicted behavior. The positive pumping component is clearly evident, indicating that the fraction of directly excited acceptors is smaller than 50%, assumed in the simulation in Figure 4, with a larger emission fraction originating from the donor energy transfer. The intensity decays of loaded NPs excited at 640 nm and observed at 820 nm do not show any anomalous intensity decay and only shortening of fluorescence lifetimes due to self-quenching.

Measured intensity decays for the donor and acceptor definitively prove the presence of energy transfer in intact NPs and allow for load estimation. We present the final experiment to demonstrate the feasibility of the proposed technology for drug delivery monitoring. NPs loaded with 66 μM were lyophilized and the stability of the NPs was monitored by DLS measurements (presented in Supplementary Table S1 and Supplementary Figure S1). Two samples were resuspended in either phosphate-buffered saline (PBS 1X) or DMSO to promote NP disintegration. In the PBS resuspension (intact NPs), we measured significant energy transfer efficiency, whereas after the NPs were resuspended in DMSO, the energy transfer disappeared, and the recovered lifetimes presented in Table 3 returning the lifetimes to the original values of donor-only and acceptor-only samples. The lifetime of the donor increased to the expected value, and the lifetime of the acceptor slightly increased to the value expected from lower concentrations and no pumping was detected. With the NP disintegration in DMSO, the donor-accepter separation became much greater in the free solution, and thus, the FRET enhancement disappeared. The heavily packed acceptor core diluted, eliminating self-quenching. The results show that the distance separation of the DiI donor and IR780 acceptors in the DMSO solution is too large to observe FRET or self-quenching. Furthermore, the significant increase in fluorescence lifetime after disintegration indicates that no complexes formed between the donor and acceptor that could lead to artificial increases in measured FRET.

Using recovered parameters for different NP sizes in Table 1 and corresponding measured donor lifetimes, we calculated the average number of chromophores loaded into the NP. For 33 μM, the transfer efficiency calculated from the average lifetime is E = 0.16, and the calculated number of chromophores in the core is 4–5 molecules. For 66 μM, the transfer efficiency is E = 0.394, which corresponds to about 14 chromophores loaded into the core, and for 99 μM, the calculated transfer efficiency is E = 0.663 which corresponds to about 34 chromophores loaded into the core. The increase pattern generally follows the acceptor concentrations used for NP preparation; however, it appears encapsulation is more efficient when increasing concentrations are used for concentrations below 100 μM.

## 3. Materials and Methods

*Materials*. Egg yolk phosphatidylcholine, free cholesterol and cholesterol ester, sodium cholate, IR780 Iodide, Dimethyl sulfoxide (DMSO), and Tris-ethylenediaminetetraacetic acid (EDTA) were obtained from Sigma Aldrich, St. Louis, MO, USA. Apolipoprotein A-I (Apo A-I) was provided by MC Labs, South San Francisco, CA, USA. DiI-C18(3) was purchased from Biotium, Fremont, CA, USA. 1,2-dioleoyl-sn-glycero-3-phosphoethanolamine-N-(carboxyfluorescein) [18:1 PE-CF] and 1,2-dimyristoyl-sn-glycero-3-phosphoethanolamine-N-[methoxy(polyethylene glycol) -1000] (ammonium salt) or 14:0 PEG 1000-PE were obtained from Avanti Polar Lipids Inc. Alabaster, AL, USA.

*Dye-Loaded Nanoparticle Preparations.* The assembly of the rHDL NPs was accomplished by a modified version of a procedure developed earlier (11). Three different formulations—(A) DiI-rHDL-IR780, (B) rHDL-IR780 (loaded control), and (C) DiI-rHDL (empty vehicle—no payload)—were prepared. Briefly, egg yolk phosphatidylcholine (EY PC) in chloroform was dried to a thin film under nitrogen. For rHDL formulations containing DiI, the desired amount of DiI in chloroform was added to the dried lipid mixture, mixed, and evaporated under nitrogen. To this lipid film, the desired amount of IR780 in DMSO was added to the vial and vortexed thoroughly. Phosphate Buffered Saline (PBS; pH 7.4) was added to the vial to yield the desired final volume of the sample before continuous vortex for 5 min. Sodium cholate (100 mM) was sequentially added to the formulations, mixed, and subsequently sonicated for 15 min (Amplitude 30/continuous mode with probe S&M 0103, Ultrasonic Processor, Newton, CT) at intervals of 3 min. The final molar ratio of Apo A-I:PC of the formulation was 1:5:1.15 M, respectively, and subsequently incubated for 24 h at 4 °C on a shaker. The sample was dialyzed (6–8 MWCO) against phosphate buffer saline (PBS; pH 7.4) for 24 h with three buffer changes in the first 6 h. The preparations were then centrifuged at 12,000 rpm for 15 min (Eppendorff Mini Spin F45-12-11, Hamburg, Germany) and filtered using a 0.22 μm syringe filter. The preparations were kept in the dark at 4 °C until further use.

*Characterization of rHDL Nanoparticles*. The dye-containing NP formulations were characterized for their physical and chemical properties. The size, zeta potential, and polydispersity index of these NPs were determined by the Malvern Zetasizer ZS90 dynamic light scattering instrument. The absorbance measurements were conducted on a Cytation 3 Imaging reader Instrument (Bio-Tek, Winooski, VT, USA).

The amount of IR780 and DiI incorporated into the NPs was estimated by absorbance measurements at 770 nm and 520 nm, respectively. The drug entrapment efficiency (*DEE*) was calculated using Equation (10).

(10) $$DEE\;\%=\left[\frac{\left(Dye\;incorporated\;into\;NP\right)}{(Initial\;Dye)\;}\right]\times 100\;$$

*Absorption and Fluorescence Measurements*. The UV–Vis absorption spectra were measured with the Cary 60 UV–Vis spectrophotometer (Agilent Technologies, Santa Clara, CA, USA). Fluorescence spectra were measured with a Cary Eclipse fluorescence spectrometer (Varian Inc., Palo Alto, CA, USA) and/or in FluoTime 300 (FT300 from Picoquant GmbH, Berlin, Germany) in square geometry setup. Both absorption and fluorescence measurements were performed using 0.4 × 1 cm quartz cuvettes with stoppers at room temperature (22 °C) and fluorescence was measured at magic angle conditions (54.7°).

*Time-resolved measurements*. The fluorescence lifetimes were measured using the FluoTime-300 high-performance fluorescence lifetime spectrometer with time-correlated single photon counting (TCSPC) module Picoharp 300 (PicoQuant, GmbH). The excitation source for lifetime measurements was 480 nm laser diode (LD), resulting in a halfwidth of the instrumental response function (IRF) of 90 ps. The DiI emission was measured at 570 nm, and IR780 emission at 820 nm was detected using an R3809U-50 micro-channel plate photomultiplier tube (MCPPMT, Hamamatsu, Inc). We also monitored intensity decays of the acceptor using a 640 nm laser diode (halfwidth of IRF ~70 ps) that only excites the acceptor. The time-resolved measurements were obtained at the magic angle condition (54.7°) to eliminate the depolarization effects due to molecular rotation or FRET. Then, the data analysis was performed using FluoFit (PicoQuant, GmbH, and Version 4.4) with a discrete multi-exponential deconvolution model (11):

(11) $$I\left(t\right)=\int_{-\infty }^{t}IRF\left(t^{\prime }\right)\sum_{i}\alpha_{i}e^{-\frac{t-t^{\prime }}{\tau_{i}}}$$

where, *IRF* (*t*′) is the instrumental response function at time *t* = *t*′, and $\alpha_{i}$ is the amplitude of the *i*th component of intensity decay at time *t* and $\tau_{i}$ is the lifetime of the ith intensity decay component. The average intensity and amplitude weighted lifetimes were calculated as follows:

(12) $$Avg\;\tau_{INT}=\sum f_{i}\tau_{i}\;\;\;\;\;\;\;\;\mathrm{Where},\;f_{i}=\frac{\sum \alpha_{i}\tau_{i}}{\sum \alpha_{i}}$$

And

(13) $$Avg\;\tau_{Amp}=\sum \alpha_{i}\tau_{i}$$

The quality of the fit to the intensity decay (lifetime) analysis was judged by the Chi-square (χ^2^) value and by the quality and autocorrelation of the residuals.

## 4. Conclusions

Herein, we demonstrate that FRET can be efficient at distances greater than R_0_ when multiple acceptors are packed in a small volume and can be used to estimate NP payload loading and stability monitoring in both storage and during delivery. For donor-acceptor systems with R_0_ = 2.8 nm (28 Å), we were able to observe high FRET efficiency for NPs with a size around 10 nm. The Forster mechanism of energy transfer was confirmed with a detailed analysis of acceptor intensity decay with a clear presence of time-dependent ‘pumping’ functions from the excited donor. It is possible to use different donors, such as Rhodamine 101 or Rhodamine 800 to significantly increase the R_0_ value and allow for load estimation for NPs up to 30 nm in diameter.

A key assumption in our analysis is that the rHDL vehicles adopt a spheroidal (loaded) or discoidal (empty) morphology, which aligns with their well-characterized structural properties. However, we acknowledge that nanoparticles can exhibit a range of morphologies beyond spherical or discoidal structures, such as elongated, rod-like, or bilayer-based structures. These structural variations may influence FRET efficiency and drug loading behavior in ways not accounted for by our current model. Future studies could explore adaptations of this method to accommodate non-spherical nanoparticle structures, particularly those resembling biological membranes or vesicular assemblies, to broaden the applicability of our approach.

We have established the presented method to estimate the chromophore/drug load for spheroidal rHDL NPs and concluded that FRET efficiency between the peripherally positioned donor molecules and core-incorporated acceptors can be used for continuous (in-line) monitoring of the integrity of the NPs and efficiency of payload release to the targeted cell. When using a near-IR emitter like IR780 such a system with long wavelength excitation in the range of 700 nm can also constitute a simple way to monitor and image dye biodistribution in tissues in real time. Additionally, this method is a convenient approach to confirm NP stability over time during storage and transport. Measured FRET efficiency also validates the diameter and the tridimensionality of the drug transporting rHDL NPs.

## Figures and Tables

**Table 1:** With constant donor (DiI) labeling, at a concentration of 214 μM, total diameter for the NPs, and approximate core size.

rHDL Acceptor Loading (μM)	Total Diameter Size (nm)	Core Size (nm) Approx.	Zeta Potential (mV)	Polydispersity Index
33	9.72 ± 0.74	3.22	−4.32 ± 0.718	0.336 ± 0.165
66	10.03 ± 1.17	3.53	−11.32 ± 0.58	0.256 ± 0.004
99	9.77 ± 0.58	3.27	−10.53 ± 1.02	0.328 ± 0.207
330	11.29 ± 0.82	4.79	−11.04 ± 3.70	0.290 ± 0.024

**Table 2:** Different acceptor (IR780) concentrations while maintaining a constant donor (DiI) concentration of 214 μM. Blank cells denoted with (---) indicates no measurement was recorded due to the properties of the sample.

		A_1_ [Cnts]	τ_1_ [ns]	A_2_ [Cnts]	τ_2_ [ns]	τ_Av 1_ [ns]	τ_Av 2_ [ns]
**Formulation**	Ex/Em						
**Donor Only**	485/570	3865.5	0.496	2196.4	1.022	0.780	0.687
**Acceptor Only**	640/820	2490.5	0.595	3988.1	0.855	0.776	0.755
33 μM Acceptor	485/570	4044.1	0.354	1786.0	0.916	0.654	0.526
	485/820	9201.2	0.877	−4100	0.240	0.965	1.388
	640/820	740.0	0.788	---	---	0.788	0.788
66 μM Acceptor	485/570	3936.1	0.251	959.8	0.770	0.473	0.353
	485/820	7944.3	0.810	-2038	0.183	0.849	1.027
	640/820	3790.9	0.636	2630.3	0.901	0.767	0.744
99 μM Acceptor	485/570	3168.2	0.198	78.64	0.991	0.286	0.217
	485/820	6958.0	0.756	−2713	0.042	0.772	1.212
	640/820	3277.9	0.565	3172.4	0.839	0.727	0.699
330 μM Acceptor	485/570	424510	0.009	49.73	1.069	0.024	0.009
	485/820	15226	0.168	3880.3	0.642	0.341	0.067
	640/820	18483	0.146	3896.6	0.628	0.375	0.230

**Table 3:** NPs loaded with 66 μM acceptors, with a constant donor concentration of 214 μM. The sample was lyophilized and resuspended in either PBS (original buffer) or DMSO (disruptive agent). Blank cells denoted with (---) indicate no measurement was recorded due to the properties of the sample.

rHDL Formulation	Average Lifetime (ns) Ex485 Em570	Average Lifetime (ns) Ex485 Em820	Average Lifetime (ns) Ex640 Em820
Donor Only	0.685	---	---
Acceptor Only	---	---	0.746
Donor + 66 μM Acceptor	0.447	0.947	0.762
PBS Resuspension (66 μM Acceptor)	0.485	0.952	0.805
DMSO Resuspension (66 μM Acceptor)	0.541	---	0.845

## Data Availability

The original contributions presented in this study are included in the article/Supplementary Material. Further inquiries can be directed to the corresponding author.

## Supplementary Materials

The following supporting information can be downloaded at: https://www.mdpi.com/article/10.3390/ijms26073276/s1.
