# Supplementary material for: Fluorescence Resonance Energy Transfer for Drug Loading Assessment in Reconstituted High-Density Lipoprotein Nanoparticles
Source: Int J Mol Sci. 2025 Apr 1;26(7):3276. doi: 10.3390/ijms26073276 (PMC11989292; doi:10.3390/ijms26073276)
Supplement: Supplementary file 1 [file ijms-26-03276-s001.zip › ijms-3522761-supplementary.pdf]

Supplemental Material

Supplemental Figures

Supplemental Table S1. The physiochemical analysis of the averages measured by dynamic light scattering (Malvern Zetasizer) for the original 66 µM acceptor loading rHDL NPs and the characteristics after lyophilizing the same formulation and reconstituting in 1X PBS.

rHDL Acceptor Loading (µM)	Size (nm)	Zeta Potential (mV)	Polydispersity Index
66 µM loading at Day 0	**10.03 ± 1.17**	**-11.32 ± 0.58**	**0.256 ± 0.004**
66 µM loading After lyophilization	**11.84 ± 1.06**	**-11.51 ± 1.21**	**0.333 ± 0.115**

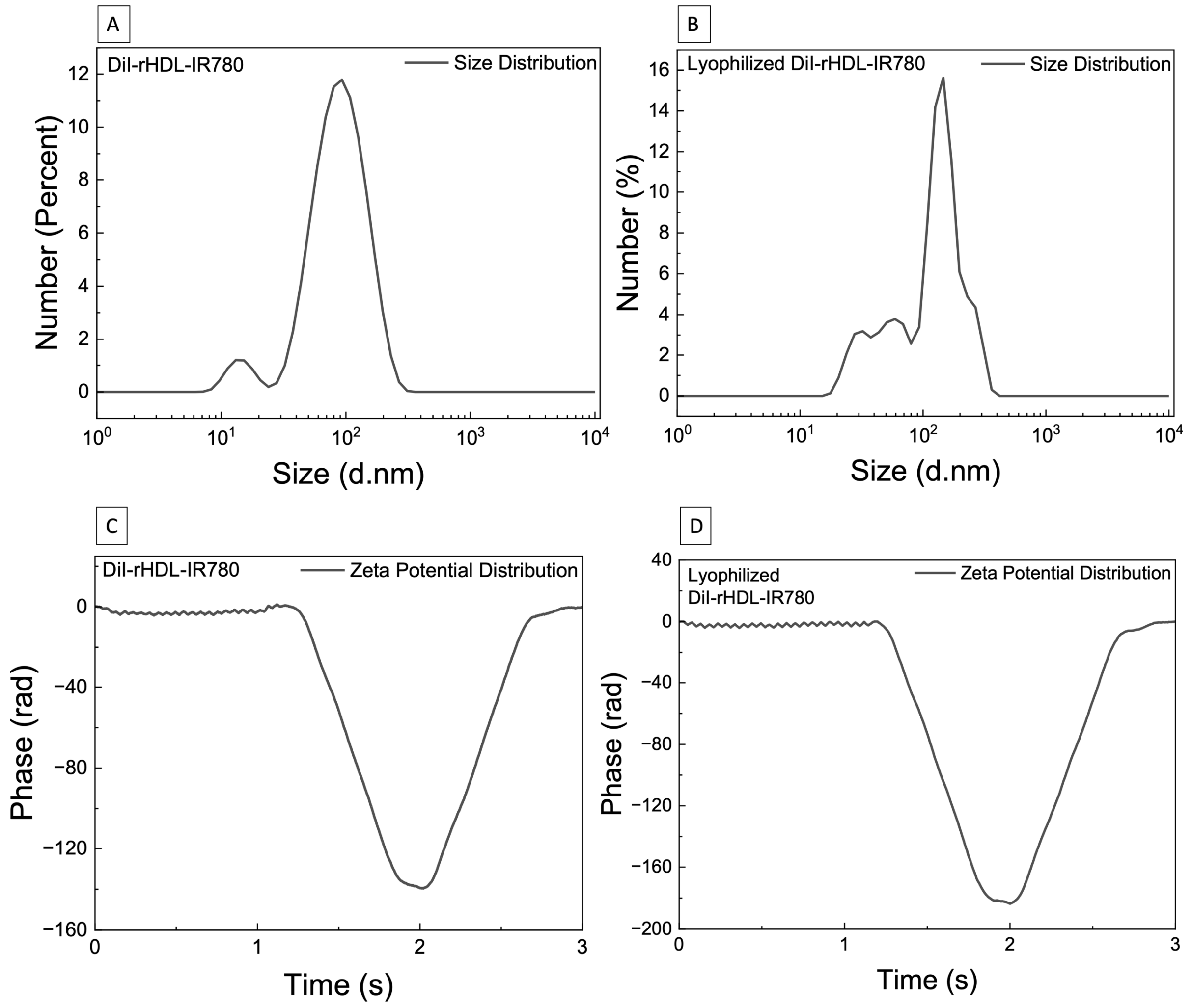

Supplemental Figure S1. The raw dynamic light scattering distribution measurements for size and zeta potential before the final calculations are made for Supplemental Table S1. **(A)** the distribution of size for the original dual fluorescent DiI-rHDL-IR780 with 66 μM loading of IR780. **(B)** the distribution of size for the dual fluorescent DiI-rHDL-IR780 with 66 μM loading of IR780 after lyophilizing and reconstituting in PBS. **(C)** the phase distribution for zeta potential for the original dual fluorescent DiI-rHDL-IR780 with 66 μM loading of IR780 before final zeta potential calculations. **(D)** the phase distribution for zeta potential for the dual fluorescent DiI-rHDL-IR780 with 66 μM loading of IR780 after lyophilizing and reconstituting in PBS before final zeta potential calculations.

Supplemental Material

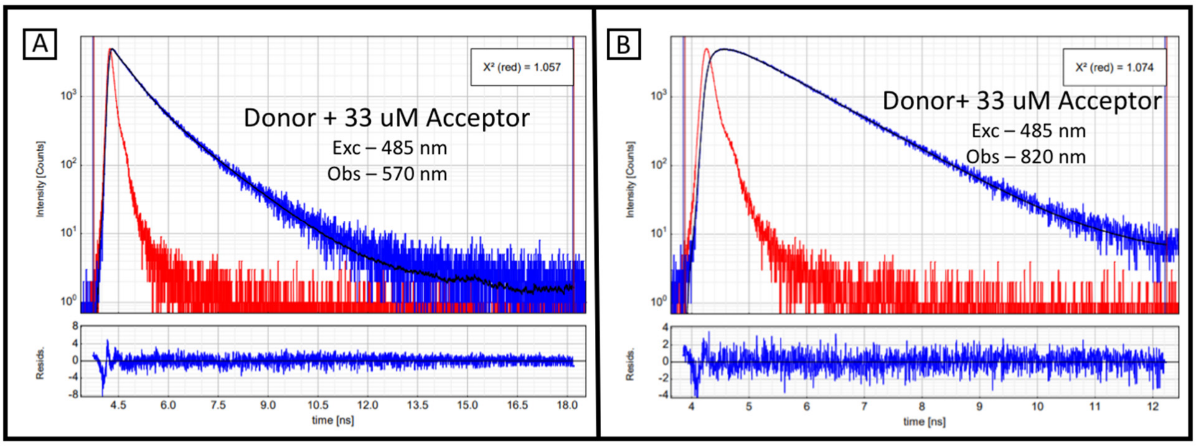

Supplemental Figure S2. (A) shows the intensity decay and average fluorescence lifetime of the rHDL NP with donor in the presence of the acceptor (33 µM loading) with excitation 485 nm and observed emission at 570 nm, (B) shows the intensity decay and average fluorescence lifetime of the rHDL NP with donor in the presence of the acceptor (33 µM loading) with excitation at 485 nm and observed emission at 820 nm.

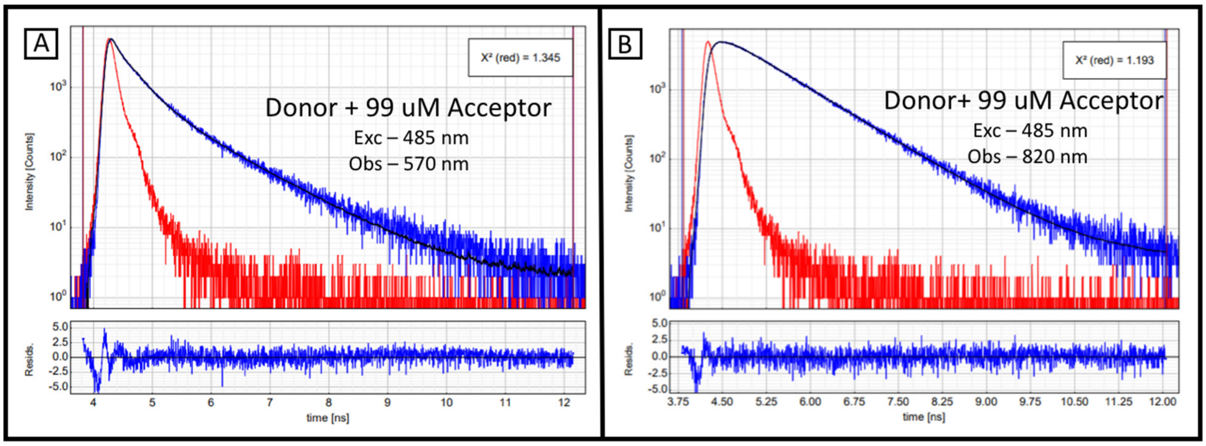

Supplemental Figure S3. (A) shows the intensity decay and average fluorescence lifetime of the rHDL NP with donor in the presence of the acceptor (99 µM loading) with excitation 485 nm and observed emission at 570 nm, (B) shows the intensity decay and average fluorescence lifetime of the rHDL NP with donor in the presence of the acceptor (99 µM loading) with excitation at 485 nm and observed emission at 820 nm.

Supplemental Material

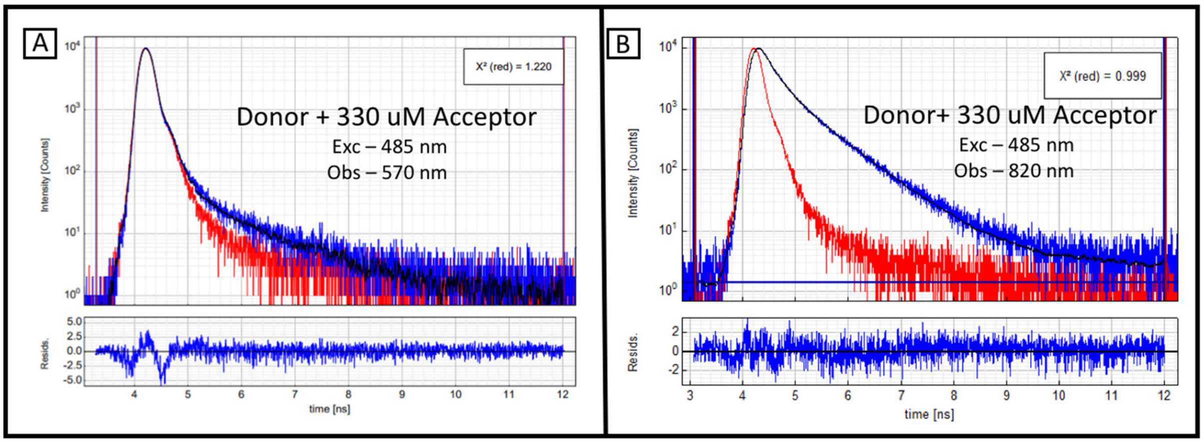

Supplemental Figure S4. (A) shows the intensity decay and average fluorescence lifetime of the rHDL NP with donor in the presence of the acceptor (330 μM loading) with excitation 485 nm and observed emission at 570 nm, (B) shows the intensity decay and average fluorescence lifetime of the rHDL NP with donor in the presence of the acceptor (330 μM loading) with excitation at 485 nm and observed emission at 820 nm.
